# Using a Herd Profile to Determine Age-Specific Prevalence of Bovine Leukemia Virus in Michigan Dairy Herds

**DOI:** 10.1155/2012/350374

**Published:** 2012-03-27

**Authors:** Ronald J. Erskine, Paul C. Bartlett, Todd M. Byrem, Chelsea L. Render, Catherine Febvay, Jessica T. Houseman

**Affiliations:** ^1^Department of Large Animal Clinical Sciences, Michigan State University, East Lansing, MI 48824, USA; ^2^Antel BioSystems, Division of NorthStar Cooperative, Inc., Lansing, MI 48909, USA

## Abstract

Enzootic bovine leukosis is a contagious disease of cattle caused by the retrovirus, bovine leukemia virus (BLV) and is the most common cause of malignant neoplasm in cattle. In order to facilitate surveillance of this disease in dairy herds, we developed a method to combine ELISA of milk collected during routine production testing with a prescribed sampling of cows that is independent of the proportion of cows within each lactation. In 113 Michigan dairy herds, milk samples from ten cows in each of the 1st, 2nd, 3rd, and ≥4th lactations were analyzed for anti-Bovine Leukemia Virus (BLV) antibodies by milk ELISA. For each herd, a BLV herd profile (BHP) was calculated as the simple average of the percent of BLV-positive cows within each of the four lactation groups. The mean BHP for all herds was 32.8%, with means of 18.5, 28.8, 39.2, and 44.8% of 1st, 2nd, 3rd, and ≥4th lactation animals infected, respectively. In eight herds, we determined the correlation between the BHP, and true herd prevalence by testing the entire lactating herd (*r* = 0.988, *P* < 0.0001). The BHP allows discrimination of lactation-specific BLV prevalence within a dairy herd, to help identify risk factors and management plans that may be important in transmission of BLV.

## 1. Introduction

Enzootic bovine leukosis is a contagious disease of cattle induced by the Δ-retrovirus, bovine leukemia virus (BLV). The disease complex is characterized by a persistent lymphocytosis which can culminate in B-cell lymphoma. Although BLV-induced lymphoma is the most common neoplastic disease of cattle, most infected cows do not display outward signs of disease, and these animals are referred to as asymptomatic or aleukemic [1]. Approximately 30–40% of BLV carriers will develop a persistent lymphocytosis while fewer than 5% develop malignant lymphosarcoma [1]. Surveys from geographic locations other than Europe have reported within-herd prevalence of BLV in adult dairy cattle from 23 to 46% [2–5].

Infection of BLV, as detected by serology, is more typical of older cattle as opposed to younger cattle [1, 6], and in one large California dairy, prevalence was reported to increase from 43% in first lactation cows to 72% in second lactation cows [7]. The effect of age distribution on herd-level BLV prevalence is so strong that differences between herds in the proportion of cows in various lactations confound any interherd comparisons of BLV prevalence. This study investigates the use of a BLV herd profile (BHP) to determine parity-related herd BLV prevalence, independent of the proportion of cows within each lactation, using a commercially available milk ELISA test. This approach provides a novel, economical, and practical method to determine herd-level BLV infection status, stratified by lactation, which can help evaluate transmission risk factors and suggest management strategies for control of BLV within a dairy herd.

## 2. Materials and Methods

### 2.1. Herd Selection

Dairy herds in Michigan, that routinely participated in Dairy Herd Improvement Association (DHIA) testing, and averaged ≥120 cows on test for the previous 12 months, were stratified into equal-sized cohorts of 119 small-sized herds (120–174 cows), 119 medium-sized herds (175–295 cows), and 119 large herds (298–6,738 cows). Within each of these strata, herds were assigned a random number which determined the order in which they were contacted and invited to participate in our study. Forty herds were sought from each cohort, but exclusions because of the inability to schedule herd visits, or lack of data within herd DHIA records, resulted in a total of 113 participating herds. The mean (±SEM) number of cows on test for each herd was 407.7 ± 67.0, and ranged from 113 to 6,492. 105 herds milked Holstein cows only, 2 herds Brown Swiss, 4 herds Jerseys, and 2 herds milked a mixture of breeds.

### 2.2. Selection of Cows for Sampling

 In order to estimate the number of cows to be sampled per herd, we calculated, by binomial proportion of standards, that if the true within-herd prevalence was 10%, there would be a 5% probability that no positive cows would be among a sample of 28 cows, that is, the probability was 95% that we would find at least one positive cow. We selected the theoretical BLV prevalence of 10% for our calculations because this is well below the mean herd prevalence reported in several studies [2–5], and thus tried to account for the difficulty to detect infected cows in herds with low prevalence. In order to improve precision, and account for attrition from the time of cow selection until actual milk sample collection, the targeted sample size per herd was increased to 40 cows. Thus, within each herd, we identified 10 cows each from the first, second, third, and ≥fourth lactations that were the most recently calved, based on current DHIA test. On the next test day for each herd, (June through August, 2010) DHIA technicians collected milk samples from the surviving selected cows for submission to the laboratory (Antel BioSystems, Inc., Lansing, MI) for ELISA testing of BLV antibodies.

### 2.3. Calculation and Validation of the BLV Herd Profile (BHP)

For each of the 113 herds, the percent of BLV-positive cows was computed for each lactation group and the percentages from all four lactation groups were averaged (with equal weight) to calculate the BHP. In order to validate the accuracy of the BHP sampling relative to total herd BLV prevalence, we stratified all 113 herds by BHP result in quartiles (very low prevalence, lower than average prevalence, above average prevalence, and very high prevalence) and randomly selected two herds from each quartile for further BLV testing. In these 8 herds, milk samples were collected from all lactating cows for BLV ELISA (*n* = 2, 158) to calculate BHP (based on 40 cows). The BHP was then compared to the average of lactation-specific prevalence (from whole-herd testing) and total herd prevalence by simple correlation analysis.

### 2.4. BLV ELISA

 Milk samples were immediately preserved with 0.2 mg/mL bronopol and 7.8 *μ*g/mL natamycin (D&F Control Systems, Inc., Dublin, CA). Samples were transported to a DHIA laboratory (Universal Laboratory Services, East Lansing, MI) and subsequently analyzed for antibodies to BLV as described below (Antel BioSystems, Inc., Lansing, MI). All transportation and storage of samples was at ambient temperature. All analyses for antibodies to BLV were conducted within 5 days of the original collection date. We previously determined that the stability of anti-*Mycobacterium avium* subsp. *paratuberculosis* antibodies in milk, stored, and detected by a commercial assay similar to the milk BLV ELISA, is consistent for 14 days at room temperature (Byrem, unpublished data). Antibodies to BLV were detected using an ultrapure virus lysate in a commercially available antibody capture ELISA (IDEXX Laboratories, Westbrook, Maine) routinely used for bulk milk analysis. Prior to analysis, individual DHIA milk samples were diluted 1 : 30 in sample diluent to reduce the effect of carryover contamination (<1%) that occurs during the DHIA sampling process. Briefly for the ELISA, antibodies to BLV in dilute milk samples bind to microtiter plate wells previously coated with virus lysate. Bound antibodies from milk are detected by reaction with horseradish-peroxidase-(HRP-) labeled monoclonal antibodies to bovine immunoglobulin (IgG). Bound HRP-labeled antibodies are detected by addition of enzyme substrate. Reaction times are standardized to the color development of positive controls (0.9 < 450 nm OD < 1.2) and stopped by the addition of 0.5 N H_2_SO_4_. Sample scores (sample OD-negative control OD) ≥0.1 were considered positive.

### 2.5. Statistical Analysis

 Correlations among herd measures of BLV prevalence were expressed as Pearson correlation coefficients (*r*). BLV prevalence rates among lactation groups were evaluated with one-way ANOVA analysis. Descriptive statistics regarding the frequency distributions, as well as all other tests described above, were done using SAS-PC (SAS Institute Inc, Cary, NC).

## 3. Results

### 3.1. BLV Index

**Figure 1:**
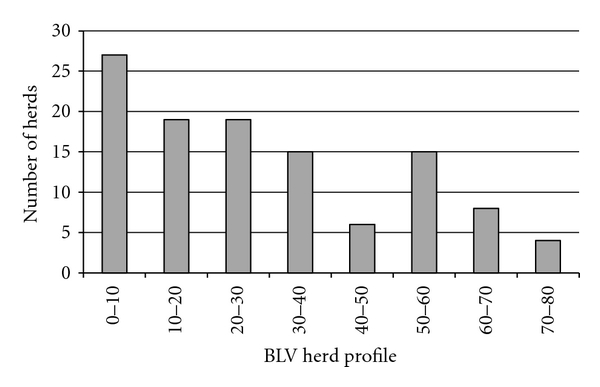
Frequency distribution of Bovine Leukemia Virus herd profile (BHP) for 113 dairy herds in Michigan. For each herd, the percent of cows that tested Bovine Leukemia Virus (BLV) positive within the first, second, third, and fourth or greater lactations (ten cows from each lactation) was averaged to calculate the BHP. The 25th, 50th, and 75th percentiles were 14.6, 30.0, and 48.9, respectively.

**Figure 2:**
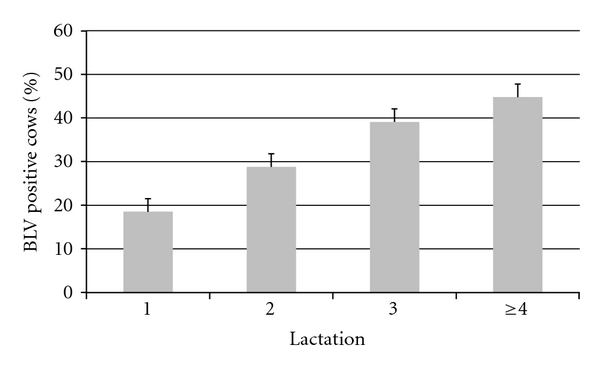
Mean prevalence, by lactation, of cows from 113 dairy herds that were positive for Bovine Leukemia Virus (BLV) by milk ELISA. For the null hypothesis that the true population correlation coefficient is zero, analysis of variance was significant (*P* < 0.0001).

**Figure 3:**
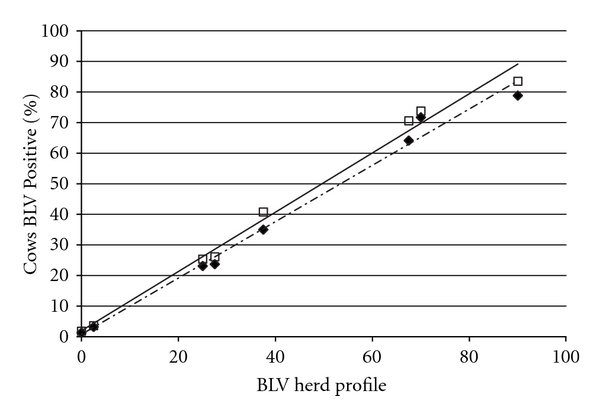
Bovine Leukemia Virus herd profile (BHP) by testing 40 milking cows (ten from each of the first, second, third and fourth or greater lactations), for Bovine Leukemia Virus (BLV) as compared to testing all milking cows in the herd and calculating either: (1) the actual BLV prevalence (solid line-□) as measured by the number of BLV positive cows divided by the total number tested, or (2) a lactation-specific BLV profile (dashed line-♦), calculated as an average of the percent of BLV infected cows in each of the four lactation groups. The correlation between the actual BLV prevalence and BHP was 0.988 (*P* = 0.0016), and the total herd profile and BHP was 0.995 (*P* = 0.0005).

 A total of 4,300 milk samples were collected from all 113 herds, from an average of 9.6 ± 0.1, 9.7 ± 0.1, 9.5 ± 0.1, and 9.2 ± 0.1 cows each from the 1st, 2nd, 3rd, and ≥4th lactations, respectively. Mean days in milk of all tested animals was 79.2 ± 0.7 days, with a median of 66 days and range of 8 to 357 days. The mean days in milk at the time of sampling for lactations 1, 2, 3, and ≥4, were 65.9 ± 0.9, 70.5 ± 1.1, 89.3 ± 1.5, and 91.0 ± 1.6 days, respectively. The mean BHP for all herds was 32.8 ± 2.1 (median of 30.0, range 0 to 80.6; Figure 1). The mean percent of 1st, 2nd, 3rd, and ≥4th lactation animals infected was 18.5 ± 2.0, 28.8 ± 2.6, 39.1 ± 2.8, and 44.8 ± 2.8, respectively (Figure 2), and differed significantly (*P* < 0.0001 by one-way ANOVA) with or without the assumption of equal variance among the 4 lactation groups. For the eight herds in which all lactating cattle were sampled and tested for BLV by ELISA, the BHP was found to be highly correlated with the actual ELISA prevalence (*r* = 0.994, *P* < 0.0001), and also highly correlated with the average lactation prevalence (*r* = 0.995, *P* < 0.0001; Figure 3).

## 4. Discussion

ELISA testing of milk for BLV antigens has been proposed as a dependable method to determine infection status [8]. We chose milk ELISA as an attractive method to coordinate large-scale herd surveillance because sample collection during DHIA testing requires little additional labor input and is more cost-effective than serum testing. In a preliminary phase of the study, we compared the ability to detect BLV antibodies by ELISA in milk as compared to serum in 142 cows from two herds (76 and 66 cows from each herd, resp.). Of the 57 serum positive cows, 49 were also milk positive (sensitivity = 86%). All 85 serum negative samples also tested negative in milk (specificity = 100%). Thus, although detecting BLV antibodies by milk ELISA was not as sensitive serum ELISA, the overall agreement between tests was >94%. Because of potential variation in BLV antibody titers in the immediate periparturient period [9], and in order to facilitate identification of enrolled cows by DHIA technicians, the second test date after calving was our targeted population of cows for sampling. We were able to sample 75% of the cows in our study by 100 days in milk, and 90% by 150 days in milk. The mean days in milk for sample collection from each cow increased with increasing lactation. This was likely due to attrition in older cows, which resulted in greater dispersal in the stage of lactation to acquire the necessary number of cows for the BHP herd profile. Further variation in days in milk at the time of sampling was caused by a small number of herds that tested every two weeks, or less frequently than monthly, and suspected cohorts of animals that calved in groups as a consequence of estrous synchronization. More consistency in stage of lactation at the time of sampling was generally obtained as herd size increased. Concern over additional sample variations relative to days in milk was our primary reason for excluding smaller herds.

The correlation between the BHP and the actual prevalence of BLV-positive cows in eight herds of diverse size and prevalence was excellent. Thus, our study suggests that the BHP provides a practical and representative herd profile that enables comparisons of BLV prevalence among herds. Additionally, by sampling the 10 most recently calved-cows, the BHP enables dairy producers to determine if heifers entering the milking herd are already BLV-positive, suggesting transmission factors specific to heifer-raising practices.

At the cow level, BLV prevalence is positively associated with lactation number (Figure 2). It is probable that age is both a cause and an effect of BLV infection. As such, it is difficult to make fair comparisons among herds with different age distributions or to monitor BLV within a herd which has a changing age distribution. By virtue of the sampling scheme within each of four lactation groups, the advantage of the BHP is that it is uninfluenced by differences or changes in the herd age distribution.

In our study, BLV prevalence was higher in older cattle. This parallels earlier reports of a higher incidence of lymphosarcoma, and positive serology, in older as compared to younger cattle [6, 7, 10]. A more recent study, using serum PCR in a dairy herd with a high prevalence of infection (85%), determined that 11%, 15%, and 24% of newborn calves, breeding age heifers, and 27-month-old cows were BLV positive, respectively [11]. Additionally, 61% of animals were infected by 36 months of age. Higher proportions of BLV seroconversion in older animals may result from longer age-associated duration of exposure to risk factors associated with the transmission of this disease, for example, use of common needles, palpation sleeves, and close contact between infected and noninfected animals [10]. However, if the ability to detect the presence of BLV in cattle by ELISA testing is limited relative to actual viral load, part of the age-associated increase in antibody prevalence, as observed in our and other studies, may be due to preexisting seronegative infections in younger cows that later seroconvert to positive status, rather than from a recent exposure to the BLV virus. This concept is refuted by previous studies of experimental BLV infections that determined serologic conversion to BLV-positive status that occurred within 3 to 14 weeks after inoculation [12–14]. However, experimental infections may not account for genetic diversity of the virus or the wide range of encountered infectious particles, that is, exposure dose, experienced during natural challenge. Several reports have identified the existence of persistently BLV-infected, as determined by PCR, but seronegative animals [15–17]. Furthermore, real-time qPCR may be more sensitive in detecting BLV proviral load in infected cattle than previously reported nested-PCR protocols [18]. If an economical method of testing for BLV by PCR can be employed in milk samples, this may provide an alternative to ELISA.

## 5. Conclusions

Overall, mean BLV prevalence of infection increased as the number of lactations increased, which likely arises from complex interactions among herd management, pathogen exposure, and possible limitations to detect all infected animals by routine screening with milk ELISA. The use of milk ELISA, coupled with employment of the BHP, enables a practical tool to determine age-stratified prevalence of BLV infection in dairy herds. We propose the BHP as a standard to help dairy producers manage BLV infection within their herds because the BHP (1) indicates which age groups are becoming infected, thereby suggesting which disease control interventions may be most effective, (2) provides a means to monitor the impact of disease control interventions, (3) allows comparisons with other herds, or historical comparisons within the same herd, regardless of differences in age distribution, and (4) is highly correlated (*r* = 0.99) with crude herd BLV prevalence.
